# Rapid Efficacy of Gemtuzumab Ozogamicin in Refractory AML Patients with Pulmonary and Kidney Failure

**DOI:** 10.3390/biology9020028

**Published:** 2020-02-10

**Authors:** Daniil Zaytsev, Larisa Girshova, Vladimir Ivanov, Irina Budaeva, Dmitri Motorin, Renat Badaev, Julia Mirolubova, Evgeni Grobovenko, Tamara Chitanava, Ekaterina Zaykova, Julia Alexeeva, Andrey Zaritskey

**Affiliations:** Almazov National Medical Research Centre, 2 Akkuratova street, 197341 St. Petersburg, Russia; lgirshova@gmail.com (L.G.); adondaron17@yandex.ru (V.I.); irina2005179@mail.ru (I.B.); dmotorin@mail.ru (D.M.); r.badaev89@gmail.com (R.B.); juli9702@yandex.ru (J.M.); e.grobovenko@mail.ru (E.G.); chitanava.tamara@yandex.ru (T.C.); catherine3452@yandex.ru (E.Z.); alexhematology@yandex.ru (J.A.); zaritskey@gmail.com (A.Z.)

**Keywords:** acute myeloid leukemia, respiratory failure, kidney failure, organ dysfunction, gemtuzumab ozogamicin

## Abstract

Objectives: To the best of our knowledge, data from Gemtuzumab ozogamicin in Acute Myeloid Leukemia (AML) patients with failure of organ functions and poor performance status are extremely lacking. Moreover, the fast recovery from organ failure, after Gemtuzumab ozogamicin administration, has never been reported. This study aimed to demonstrate the efficacy and rapid response of Gemtuzumab ozogamicin in refractory acute myeloid leukemia (AML) patients with pulmonary and kidney failure and poor performance status. Three refractory AML patients, with organ dysfunction, are described. One patient was pre-treated with intensive chemotherapy, and two other patients progressed during Azacitidine treatment. Two patients had respiratory failure grade 2 and one patient suffered from acute kidney insufficiency. Two patients were highly febrile with an elevated С-Reactive Protein (CRP) level. The WHO performance status of three was measured in all patients. Gemtuzumab ozogamicin administration was performed in three patients, followed by a further switch to Gemtuzumab ozogamicin + Azacitidine or “7+3” treatment. Results: Gemtuzumab ozogamicin administration resulted in abrupt fever cessation in two febrile patients simultaneously with a rapid decrease in CRP level and fast resolution of respiratory failure. Recovery of kidney function was noticed rapidly in patients with renal insufficiency. The WHO performance status was elevated in all three patients. No adverse grade II–III effects were noticed. Further treatment made two patients eligible for intensive chemotherapy, one patient underwent allogeneic stem cell transplantation, and the patient with kidney failure obtained complete remission. Conclusions: Gemtuzumab ozogamicin therapy appeared to be safe and highly efficacious in relapsed/refractory AML patients with organ dysfunction, like pulmonary or renal failure and poor performance status, and may contribute to rapid recovery from organ failures.

## 1. Introduction

Patients with acute myeloid leukemia (AML) comprise the cohort of patients at a higher risk of life-threatening complications and intensive care unit (ICU) admission for intensive monitoring and treatment due to the severity of the disease, hospital-acquired infections, and intensive chemotherapy regimens used [1,2].

Relapsed and refractory AML (10–40% of AML) represents the most common group of AML patients with organ dysfunction and poor outcomes [3].

Low toxicity of Gemtuzumab ozogamicin (GO) seems to provide a new, promising option for the treatment of highly compromised patients. Amadori et al. reported the results of the GIMEMA trial of GO versus best supportive care in the treatment of unfit for intensive chemotherapy patients in a front-line setting [4]. The toxicity of GO was comparable to best supportive care, whereas a statistically significant increase in overall survival was shown in the GO arm. Moreover, GO has been shown to be an efficacious treatment in relapse/refractory AML patients [5,6]. However, significant organ dysfunction was the exclusion criterion in all these trials. To the best of our knowledge, data of GO in AML patients with organ failure are lacking.

Here, we describe three patients with refractory CD 33+ AML and organ failures who benefited from GO use. All of them had uncontrolled leukemic overgrowth. The WHO performance status got progressively worse during prior treatment. 

Most illustrative clinical data and the results of the therapy of patient No. 1 are presented below. Clinical data and results of the therapy of patient No. 2 and No. 3 are summarized in Table 1.

## 2. Clinical Cases

The 35-year-old male patient with primary chemorefractory acute myeloid leukemia with maturation was designated as an intermediate risk group (ELN 2017). Bone marrow was hypercellular with 33.5 % of blast cells. Cytogenetic analysis revealed a trisomy 8. No molecular abnormalities were detected.

Two induction cycles of «7+3» regimen without remission achievement were followed by therapy in “FLAG” regimen. The latter therapy was complicated with a febrile neutropenia, the bloodstream infection associated with Ralstonia pickettii and polyresistant Klebsiella pneumoniae, an activation of cytomegaloviral infection. Antibacterial medications, according to in vitro sensitivity and empirical antimycotic therapy, were started on day 8 of “FLAG”. Ganciclovir was added on day 9 of “FLAG”. Four consecutive switches of antibacterial therapy and two switches of antifungal therapy were made. The latest modification of antifungal therapy was made on day 13 and antibacterial therapy on the following day. During the whole period of antibacterial/antifungal therapy, the patient condition continued to get worse. The patient had experienced high fever, and CRP increased to 295 mg/L. The WHO performance status of three was assessed. Eventually, the patient had progressed to acute respiratory failure grade two with progressive increasing of dyspnea and worsened hypoxemia. 

An increase in the size of lung infiltrates was revealed on serial CT scans.

**Figure 1:**
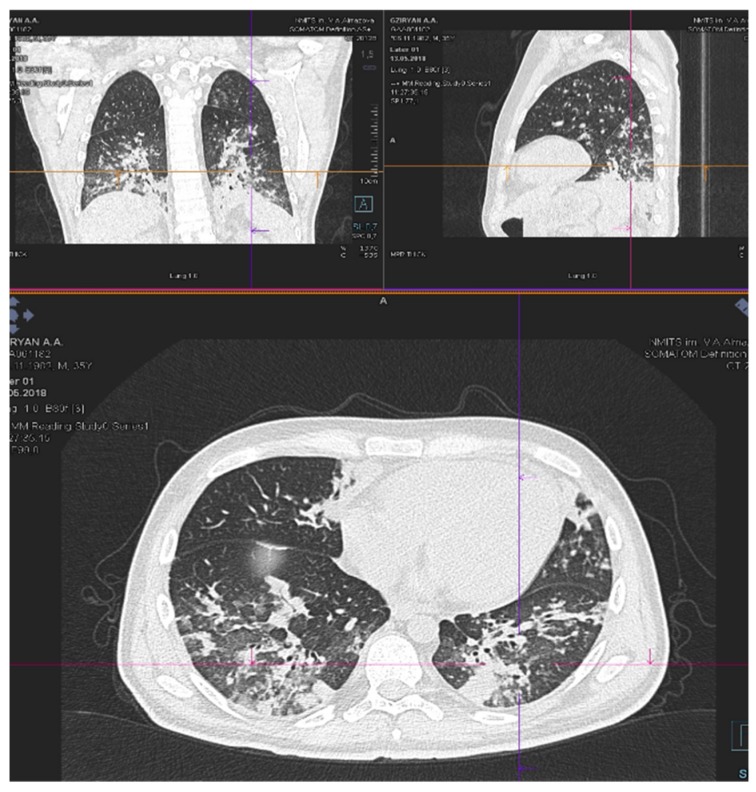
CT chest scan on day 18 of “FLAG”.

A chest CT scan on day 18 of “FLAG” showed massive infiltrates in the basal segments of the lower lobes in both lung fields with dense peri-bronchial lesions and multiple small interstitial lesions in segments on both sides of the lungs (Figure 1).

Repeated bronchoalveolar lavages were negative for any pathogens, including fungi and viruses and galactomannan. 

Marrow blast cells reached 91.6% with 62.2% of CD33 positive cells on day 19 of “FLAG”.

Gemtuzumab ozogamicin therapy was started on day 20 of “FLAG” therapy (Day 1 of GO).

During Day 1 after GO administration blood gas normalization with acute respiratory failure recovery was achieved. Apyrexia was noticed on Day 2 after GO infusion.

**Figure 2:**
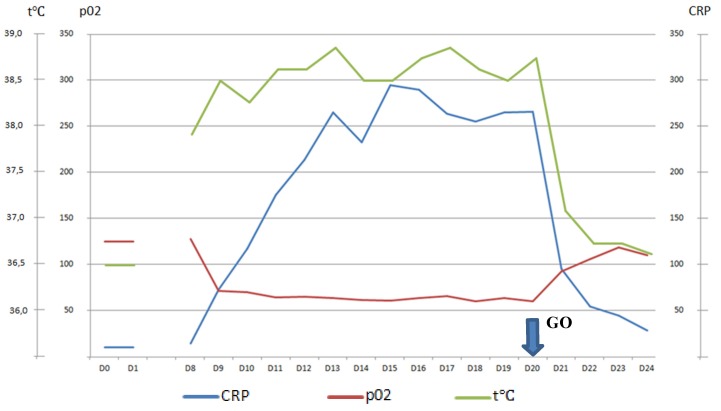
CRP, pO2, temperature after “GO” Day20–Day 24.

CRP started to decline immediately and fell to 29 mg/L on Day 5 after GO administration (Figure 2).

The WHO performance status improved to grade 2.

**Figure 3:**
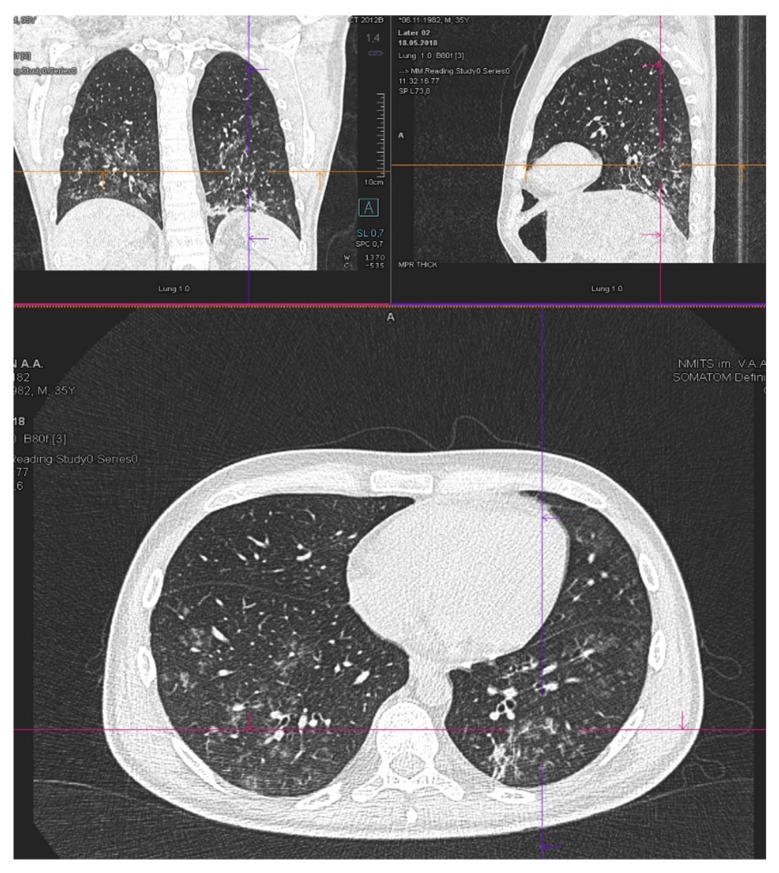
CT chest scan on day 3 of “GO”.

A chest CT scan on Day 3 of GO therapy showed a significant regression of pulmonary infiltrates in size and their density (Figure 3).

Therapy was augmented by «Gemtuzumab ozogamicin + Azacitidine» regimen (GO 3 mg/m^2^ day 8; azacitidine 50 mg/m^2^ days 1–7), which was started on Day 5 after GO. The patient was treated by the two consecutive cycles of the therapy in «Gemtuzumab ozogamicin+Azacitidine» regimen. No non-hematological adverse grade 3–4 effects were observed. Antibacterial therapy was gradually de-escalated and stopped. Marrow blast cell count in the marrow gradually decreased. The best response after the second cycle of therapy was morphologic leukemia-free state (MLFS) achievement: Blast count was 1.5% without peripheral blood cells recovery (not shown).

The patient underwent allogeneic stem cell transplantation from a match-related donor with complete remission and donor chimerism achievement (not shown).

## 3. Discussion

In three patients with primary resistance to standard chemotherapy regimens (“FLAG” or azacitidine), poor performance status and respiratory or acute kidney failure, GO monotherapy was treatment due to the primary resistance and poor performance status. At the next step, two of our patients were switched to combined Gemtuzumab ozogamicin plus Azacitidine (GO+Aza) treatment due to the ability of the latter to potentiate the efficacy of GO and overcome resistance to GO [6,7]. CD33 blast cell positivity was obligatory for including the patients in this treatment plan.

In patient with acute kidney injury with no prior history of chronic kidney disease, dehydration, use of nephrotoxic agents or tumor lysis syndrome signs (potassium, calcium, phosphorus, uric acid were unremarkable), creatinine started to decrease only after GO and contemporaneously with blast cells clearance. Patients with respiratory dysfunction and leukemia progression were resistant to preceding (at minimum six days) and ongoing antimicrobial/antiviral therapy. Following GO administration, their arterial blood gases gradually improved over the first days and apyrexia was achieved in one to two days after GO.

Therefore, a rapid resolution of renal insufficiency on GO therapy (patient No. 2), as well as a drastic decrease of infiltrative lung lesions (patient No. 1 and No. 3) could presumably be attributed to the anti-leukemic effect of this medication. This situation is not rare, and the overall incidence of extramedullary lesions reported in the literature range from 2.5% to 30% [8]. Unfortunately, due to thrombocytopenia, grade 4 with refractoriness to platelets transfusion organ biopsy was not performed in all our patients.

The response to mono-GO, or in combination with azacitidine, that has been previously described in relapsed and refractory AML patients, even with extramedullary involvement [9,10,11], the immediate response to GO in critically ill patients with pulmonary or renal failure has not been reported. Nevertheless, the mechanism of GO pharmacokinetics provides some explanation for this prompt effect. The GO rapidly binds to its receptor, followed by the immediate internalization of cytostac-antibody complex, leading to apoptosis of CD33 positive leukemia [12].

The alternative explanation of the rapid response to GO therapy, which can lead to recovery from organ dysfunction, could result from its significant immunomodulatory effect.

In a xenomodel of macrophage activation syndrome, GO resolved the symptoms completely, and led to complete recovery of experimental animals [13]. Pathogenetic mechanisms of sepsis and macrophage activation syndrome are close [14]. The rapid efficacy of GO in our patients possibly arises from its ability to decrease the level of pro-inflammatory cytokines through CD33 positive cells elimination, such as CD33 positive blast cells, neutrophils, and monocytes, thereby, ameliorating the clinical and laboratory signs of tissue damage. Moreover, the incidence of grade 3 or 4 sepsis (17%) and pneumonia (8%) was lower than expected in relapsed AML CD33 positive patients treated with GO [15]. It is highly likely rapid apyrexia is associated with GO therapy through IL-6 declining, being one of the most important factors trigged a febrile response [16], as all our patients who had fever antimicrobial therapy had not been changed for at least six or seven days. On the other side, a concomitant reduction of pulmonary lesions seems to explain a fever cessation through direct anti-leukemic effects of GO.

The limitations in this case report, includes a lack of cytokine-level data and morphological assessment of organ lesions, which could strongly confirm our interpretation.

Nevertheless, we suggest Gemtuzumab ozogamicin could be safe and highly efficacious in relapsed/refractory AML patients with poor performance status and organ dysfunctions.

## 4. Conclusions

We made a successful attempt to treat three severely ill patients with respiratory failure and kidney insufficiency by GO. In all of the patients, respiratory failure and acute kidney injury resolution was achieved in a few days after a single GO infusion. To the best of our knowledge, it is the first case report, describing a rapid response, which has found abrupt fever cessation with CRP decreasing and recovery from respiratory or kidney failure after GO administration. No additional hematological or non-hematological toxicity was noticed. 

## Figures and Tables

**Table 1:** Summary of clinical data and results of the therapy of patient No. 2. and No. 3.

Sex Age	Male Patient. 74 Years Patient No. 2	Male Patient. 54 Years Patient No. 3
**Diagnosis**	AML without maturation (NOS, WHO 2016). Intermediate risk group (ELN2017). Normal karyotype. Primary refractory disease.	AML with maturation (NOS, WHO 2016). Add (21) (q22). Intermediate risk group (ELN2017). Primary refractory disease.
**Clinical data**	A patient with a progression disease during the treatment by azacitidine. Increasing blast cells in blood and marrow by more than 50% from baseline after 2 cycles of azacytidine therapy.	A patient with a progression disease during the treatment by azacitidine. Increasing blast cells in blood and marrow by more than 50% from baseline after 2 cycles of azacytidine therapy.
**Patient status before GO therapy initiation**	The WHO performance status of 3. Marrow blast cells 88.6%, peripheral blood blast cells 60%, pancytopenia grade 3–4. CRP was slightly increased to 20 mg/L. Acute kidney failure grade 2 with no prior history of chronic kidney disease dehydration, use of nephrotoxic agents or tumor lysis syndrome signs. Creatinine increasing up to 2.8xULN and GFR decline to 15 mL/min.	The WHO performance status of 3. Marrow blasts cells 68%. High fever and elevated CRP level up to 332 mg/L with no response to escalated antibiotics/antimycotics combination. The patient had respiratory failure grade 2 with massive bilateral polysegmental lung infiltrates according to a chest CT scan.
**Regimen of therapy with GO**	«GO » 1 cycle «GO+Aza» 1 cycle	«GO » 1 cycle
**Response to therapy**	The WHO performance status improved to grade 2.	The WHO performance status improved to grade 2. Apyrexia was achieved on day 3 of the GO therapy.
	Kidney function began to improve immediately after GO infusion. Creatinine started to decrease on day 1 of the therapy and returned to normal value on day 6 (GFR elevated up to 72 mL/min on day 6). Thus, recovery after acute kidney injury occurred on day 6. There was blast clearance in peripheral blood on day 5 after GO therapy.	CRP level started to drop on day 1 of the therapy (CRP on day 2—250 mg/L, on day 7—60 mg/L)
	On day 5 after GO infusion «GO+Aza» therapy was initiated. No laboratory signs of kidney injury were noticed during whole period of therapy in the «GO+Aza» regimen.	A chest CT scan on day 6 of the GO therapy showed a significant regression of pulmonary infiltrates in the size.
	Day 14 marrow blast cells 16.1%.	Day 7 marrow blast cells 46%. Thus, blast cell reduction was achieved on day 7 after GO infusion.
	Peripheral blood cell recovery was achieved on day 40 of the «GO+Aza» therapy. On day 40 the marrow blast cell was 1%. Thus, complete remission with peripheral blood cell recovery was achieved.	Patient became eligible for chemo intensification. On day 12 of the therapy, “7+3” was initiated.
